# Dynamics of Islet Autoantibodies During Prospective Follow-Up From Birth to Age 15 Years

**DOI:** 10.1210/clinem/dgaa624

**Published:** 2020-09-03

**Authors:** Petra M Pöllänen, Samppa J Ryhänen, Jorma Toppari, Jorma Ilonen, Paula Vähäsalo, Riitta Veijola, Heli Siljander, Mikael Knip

**Affiliations:** 1 Pediatric Research Center, Children’s Hospital, University of Helsinki and Helsinki University Hospital, Helsinki, Finland; 2 Research Program for Clinical and Molecular Metabolism, Faculty of Medicine, University of Helsinki, Helsinki, Finland; 3 Department of Pediatrics, Turku University Hospital, and Institute of Biomedicine and Centre for Population Health Research, University of Turku, Turku, Finland; 4 Immunogenetics Laboratory, Institute of Biomedicine, University of Turku and Clinical Microbiology, Turku University Hospital, Turku, Finland; 5 Department of Pediatrics, PEDEGO Research Group, Medical Research Center, Oulu University Hospital and University of Oulu, Oulu, Finland; 6 Tampere Center for Child Health Research, Tampere University Hospital, Tampere, Finland; 7 Folkhälsan Research Center, Helsinki, Finland

**Keywords:** type 1 diabetes, prediction, children, HLA, islet autoantibodies, dynamics

## Abstract

**Context:**

We set out to characterize the dynamics of islet autoantibodies over the first 15 years of life in children carrying genetic susceptibility to type 1 diabetes (T1D). We also assessed systematically the role of zinc transporter 8 autoantibodies (ZnT8A) in this context.

**Design:**

HLA-predisposed children (N = 1006, 53.0% boys) recruited from the general population during 1994 to 1997 were observed from birth over a median time of 14.9 years (range, 1.9-15.5 years) for ZnT8A, islet cell (ICA), insulin (IAA), glutamate decarboxylase (GADA), and islet antigen-2 (IA-2A) antibodies, and for T1D.

**Results:**

By age 15.5 years, 35 (3.5%) children had progressed to T1D. Islet autoimmunity developed in 275 (27.3%) children at a median age of 7.4 years (range, 0.3-15.1 years). The ICA seroconversion rate increased toward puberty, but the biochemically defined autoantibodies peaked at a young age. Before age 2 years, ZnT8A and IAA appeared commonly as the first autoantibody, but in the preschool years IA-2A– and especially GADA-initiated autoimmunity increased. Thereafter, GADA-positive seroconversions continued to appear steadily until ages 10 to 15 years. Inverse IAA seroconversions occurred frequently (49.3% turned negative) and marked a prolonged delay from seroconversion to diagnosis compared to persistent IAA (8.2 vs 3.4 years; *P* = .01).

**Conclusions:**

In HLA-predisposed children, the primary autoantibody is characteristic of age and might reflect the events driving the disease process toward clinical T1D. Autoantibody persistence affects the risk of T1D. These findings provide a framework for identifying disease subpopulations and for personalizing the efforts to predict and prevent T1D.

Before the manifestation of clinical type 1 diabetes (T1D), autoantibodies against islet autoantigens are usually detected in the circulation of prediabetic individuals. They are considered to reflect ongoing disease activity and are useful tools for identifying individuals at risk for T1D (1). The disease risk is notably increased in the presence of 2 or more biochemical autoantibodies when compared to rather nonprogressive islet autoimmunity characterized by positivity for a single autoantibody (2, 3). More than 80% of multipositive individuals progress to clinical diabetes in 15 years, although the pace of progression is highly variable (2). Autoantibody titer and affinity, and different antibody combinations, have been observed to increase the prognostic value of autoantibody testing in the prediction of T1D (3-5).

Both genetic predisposition, mainly determined by the human leukocyte antigen (HLA) class II genes, and environmental factors, play an important role in the disease process leading to autoimmunity and eventually to clinical disease (6). Genetic polymorphisms both in HLA and non-HLA genes contribute to the initiation of autoimmunity as well as to the progression to overt diabetes (7). The disease process beginning usually with either autoantibodies to insulin (IAA) or to glutamic acid decarboxylase (GADA) as the first autoantibody shows different characteristics of islet autoimmunity and genetic associations, implicating heterogeneity of the disease process (8-12). The primary autoantibody signature might predict the disease progression and the timing of the disease presentation.

At present, the screening for HLA-conferred disease risk and islet autoantibodies provides the most reliable and accessible means to stratify children into risk categories for T1D and to identify individuals for future intervention trials aimed at preventing or delaying the onset of overt disease (3). However, despite extensive research efforts autoantibody testing and genetic screening are far from being a perfect tool for assessing individual diabetes risk or the timing of the manifestation of clinical disease. Accordingly, there is a need for more information on the natural dynamics of disease-associated autoantibodies in childhood to better distinguish between nonprogressive immunological activation and true progressive autoimmunity, and to further elucidate the mechanisms underlying the heterogeneity of the disease process.

In this prospective study we characterized the dynamics of islet autoantibodies during the first 15 years of life. We have previously reported seroconversion dynamics of diabetes-associated autoantibodies by ages 2 and 5 years in the same study cohort (13, 14). In the present analysis we put special emphasis on age-dependent differences in the seroconversion rate of individual autoantibodies, the predictive value of autoantibody testing for clinical T1D, the role of primary autoantibody signatures in disease progression, and the possible disappearance of autoantibodies during childhood. We also examined the value of islet cell antibodies (ICA) in disease prediction as a part of the autoantibody repertoire and assessed systematically the role of zinc transporter 8 autoantibodies (ZnT8A) in this context.

## Materials and Methods

### Study participants and their samples

The study individuals were participants in the Finnish population-based Type 1 Diabetes Prediction and Prevention (DIPP) study that aims at monitoring the appearance of islet autoantibodies in children with increased HLA-conferred susceptibility to T1D and at identifying means to prevent or delay the onset of overt disease in at-risk individuals. The DIPP Study is an ongoing observational birth cohort study in the Turku, Oulu, and Tampere University Hospitals. The study protocol has been described in detail in our earlier reports (3, 15). In brief, the children were seen every 3 to 6 months during the first 2 years and every 6 to 12 months thereafter, unless they turned autoantibody positive, in which case monitoring continued every 3 months. The first study visit took place at age 3 months. Every effort was made to minimize the dropout rate during the 15-year follow-up. At clinical visits, local anesthesia creams were applied on skin before blood sampling to prevent discomfort and pain. If a participant missed an appointment, the families were encouraged to contact the research nurse to reschedule the visit. The study visits were free of charge.

In the present study, the first 1006 children (53.0% boys) recruited to the DIPP Study, born between November 1994 and July 1997, were observed up to a maximum age of 15.5 years for the development of ICA, IAA, GADA, ZnT8A, and autoantibodies to islet antigen-2 (IA-2A). Follow-up visits taking place after age 14.5 years were considered as the final 15-year visits. Autoantibody samples obtained before age 15.5 years were included in the detailed autoantibody analyses.

### Genetic screening

The eligible participants carried either the high-risk *HLA* genotype DQB1*02/*0302 or the moderate-risk genotypes DQB1*0302/x (x≠*02, *0301, or *0602) (16, 17). Screening for the *HLA-DR/DQ* risk haplotypes was performed on cord blood by using polymerase chain reaction amplification followed by time-resolved fluorometry (16-18).

### Autoantibody analyses

The diabetes-associated autoantibodies were analyzed in the Research Laboratory, Department of Pediatrics, University of Oulu, Oulu, Finland, except for ZnT8A, which were analyzed in the PEDIA laboratory, University of Helsinki, Helsinki, Finland. ICA were analyzed using an indirect immunofluorescence staining method, as previously described (19). The detection limit for ICA positivity was 2.5 Juvenile Diabetes Foundation units (JDFU). Standardization of the ICA assay has been described in the Supplemental Methods (20). The biochemical autoantibodies IAA, GADA, IA-2A, and ZnT8A were measured with specific radiobinding assays, described in detail previously (21-25). Study samples with autoantibody titers between the 97th and 99.5th percentile values of the reference population comprising 370 to 374 nondiabetic Finnish children were reanalyzed to confirm the result. Based on the results from the Diabetes Autoantibody Standardization Program and the Islet Autoantibody Standardization Program in 2010 to 2016, the sensitivities of the IAA, GADA, IA-2A, and ZnT8A radiobinding assays have been 36% to 62%, 64% to 88%, 62% to 72%, and 62% to 70%, respectively, and the corresponding specificities have been 94% to 98%, 94% to 99%, 93% to 100%, and 99% to 100%, respectively.

### Definitions

Seroconversion to confirmed autoantibody positivity was defined as testing positive for the autoantibody in at least 2 consecutive samples. The date of seroconversion was defined as the date of draw of the first autoantibody-positive sample. Multipositivity was defined as simultaneous positivity for at least 2 autoantibodies in 2 consecutive samples. Only autoantibodies turning positive before the diagnosis of clinical T1D were included in the detailed autoantibody analyses. Infants with maternal autoantibodies were excluded from the autoantibody analyses if no de novo production of islet autoantibodies was detected. T1D was diagnosed according to the World Health Organization criteria (26). Progressors were defined as individuals who had been diagnosed with T1D. Inverse seroconversion was defined as turning permanently autoantibody negative after testing autoantibody positive in at least 2 consecutive samples. Fluctuating autoantibody positivity was defined as 1 or more autoantibody negative samples between positive samples, that is, at least 2 consecutive positive samples before and after the negative samples. Autoantibody titers were compared among individuals positive for the specific autoantibody reactivity.

### Data analysis

IBM SPSS Statistics 25.0 statistical software for MacIntosh was used for multiple parametric and nonparametric statistical analyses. The CI was set at 95% and the 2-tailed statistical significance was set at *P* less than .05. Cross-tabulation, the Pearson χ ^2^ test, the Fisher exact test, the Mann-Whitney *U* test, and the Kruskal-Wallis test were applied to test statistical differences, when applicable. Survival distributions were tested by using the log-rank test. Survival tables were created by using the GraphPad Prism 8 software for Mac OS X. Sensitivity and specificity values were determined as previously described (27), and the corresponding 95% CIs were calculated by using a web-based statistical software (http://statpages.info/ctab2x2.html). Missing data were excluded from the analyses. Corrections for multiple testing were not applied in the present data analysis because of the overly conservative nature of the Bonferroni correction. The achieved results were, however, interpreted cautiously, taking into account that the null hypotheses may have been falsely rejected by chance because of the multiple testing.

### Ethical considerations

The legal representatives of the study participants have given written informed consent for HLA-screening and for participation in the DIPP Study. The study protocol has been approved by the local ethics committees of the 3 participating hospitals, and the study has been carried out according to the principles of the Declaration of Helsinki as revised in 2008.

## Results

### Description of the study cohort

**Figure 1.**
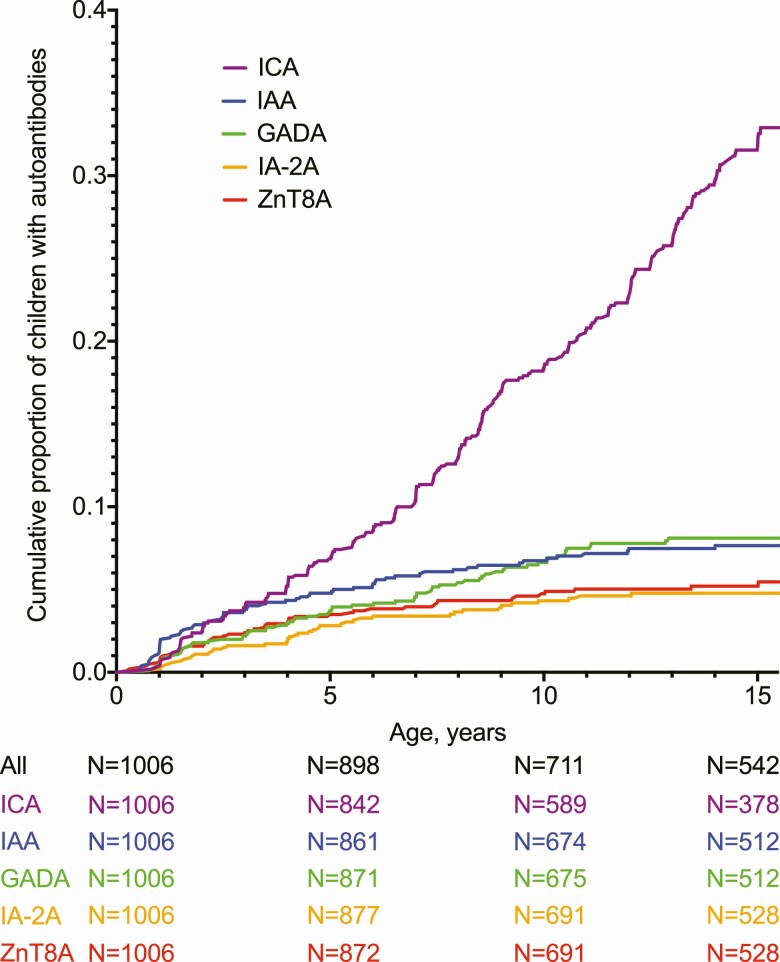
Development of islet cell (ICA), insulin (IAA), glutamate decarboxylase (GADA), islet antigen-2 (IA-2A), and zinc transporter 8 (ZnT8A) antibodies by age 15.5 years.

The study participants were observed from birth over a median time of 14.9 years (range, 1.9–15.5 years), including progressors to T1D (20). Altogether 542 (55.8%) nonprogressors completed the 15-year follow-up (Fig. 1). By age 15 years, 35 (3.5%) children had progressed to T1D. Altogether 275 (27.3%) children had confirmed seroconversion to autoantibody positivity, including 32 progressors. In addition, 2 progressors had their first autoantibody-positive sample at diagnosis. Both of them had dropped out from the autoantibody follow-up several years before the diagnosis (3.6 and 10.4 years). By age 15 years, the relative proportions of the HLA risk groups remained essentially unaltered, with 136 children (25.1%) in the high-risk and 406 (74.9%) in the moderate-risk group. The proportion of progressors was higher in the high-risk than in the moderate-risk HLA group (8.3 vs 1.9%; *P *< .001). Although there was no difference in the sex distribution between progressors and nonprogressors, a higher proportion of progressors were boys after age 10 years compared to younger progressors (n = 10, 83% vs n = 9, 39%; *P* = .03). At birth, 5 (14%) progressors and 31 (3%) nonprogressors had a first-degree relative affected by T1D (20).

### Appearance of diabetes-associated autoantibodies

**Figure 2.**
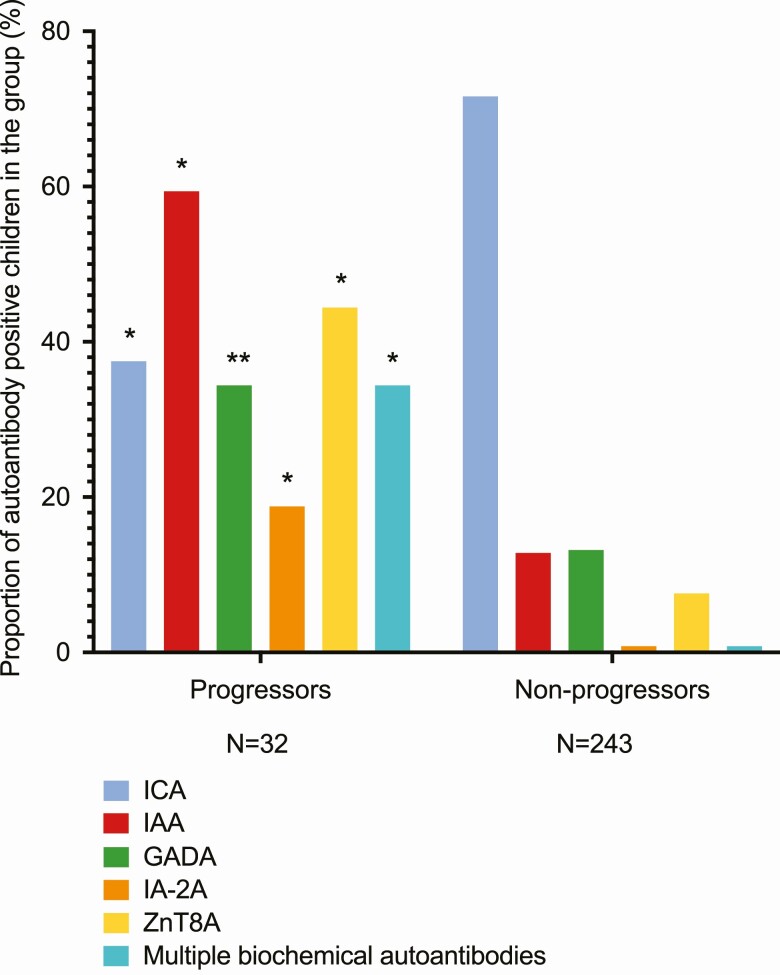
Autoantibodies at initial seroconversion among progressors and nonprogressors. *P less than .001. **P less than .002.

The overall seroconversion rate increased over the first 15.5 years, mostly because of ICA seroconversions (Table 1). When ICA was omitted from the screening repertoire, the age at seroconversion decreased (20). Children carrying the high-risk HLA genotype developed autoantibodies more frequently than those with moderate-risk genotypes (36 vs 24%; *P *< .001; screening without ICA 18 vs 9%; *P *< .001). Among the seroconverted children, we observed no statistically significant differences in the frequency of ZnT8A, IA-2A, or ICA positivity between the HLA genotypes, but the development of IAA and GADA positivity showed higher frequency among those with the high-risk genotype (data not shown). There were no sex differences in the overall seroconversion rate when approaching puberty (data not shown). Autoantibody characteristics among the progressors and nonprogressors are presented in Fig. 2 and Supplemental Table 2 (20).

**Table 1.** Cumulative frequencies of autoantibodies by age 15.5 years, median age at seroconversion, and seroconversion rates in 4 age periods (0-1.99, 2-4.99, 5-9.99, and 10-15.5 years)

Autoantibody type	Cumulative frequency of autoantibody at 15.5 y, %^*a*^	Median age at seroconversion (range), y	Seroconversions/100 follow-up, y			
			0-1.99	2-4.99	5-9.99	10-15.5
ICA ≥ 2.5 JDFU	245 (24.4)	8.1 (0.5-15.1)	1.2	1.5	2.3	2.8
ICA ≥ 10 JDFU	72 (7.2)	4.2 (0.5-14.5)	1.1	0.6	0.5	0.4
ICA ≥ 20 JDFU	50 (5.0)	4.2 (0.8-12.7)	0.7	0.5	0.4	0.2
IAA	69 (6.9)	2.5 (0.5-14.0)	1.4	0.6	0.4	0.2
GADA	69 (6.9)	5.0 (0.8-12.9)	0.9	0.6	0.6	0.3
IA-2A	42 (4.2)	4.1 (1.0-12.1)	0.5	0.6	0.3	0.1
ZnT8A	48 (4.8)	3.1 (0.3-15.0)	0.8	0.6	0.3	0.1
At least 1 autoantibody	275 (27.3)	7.4 (0.3-15.1)	2.0	1.8	2.5	2.7
At least 1 autoantibody, ICA ≥ 10 JDFU	126 (12.5)	4.0 (0.3-14.5)	2.0	1.2	0.9	0.5
At least 1 autoantibody, ICA ≥ 20 JDFU	113 (11.2)	4.0 (0.3-14.0)	1.7	1.0	0.9	0.5
At least 1 biochemical autoantibody	113 (11.2)	4.0 (0.3-14.0)	1.7	1.1	0.9	0.5
Multiple (≥ 2) autoantibodies	71 (7.1)	4.0 (0.8-14.4)	1.1	0.6	0.5	0.4
Multiple biochemical autoantibodies	50 (5.0)	3.0 (0.8-12.1)	1.0	0.5	0.3	0.1

Abbreviations: IAA, insulin autoantibodies; IA-2A islet antigen-2 autoantibodies; ICA, islet cell antibodies; GADA, glutamate decarboxylase autoantibodies; JDFU, Juvenile Diabetes Foundation units; ZnT8A, zinc transporter 8 autoantibodies.

^*a*^Proportion of children in study population (N = 1006).

### The dynamics of islet autoantibodies

All antibodies demonstrated increasing cumulative proportions up to age 15 years (see Fig. 1). To assess the dynamics of autoantibodies, we calculated seroconversion rates in the age periods 0 to 2, 2 to 5, 5 to 10, and 10 to 15.5 years (Table 1). The primary ICA seroconversion rate increased continuously toward puberty, but primary seroconversions positive for IAA and ZnT8A peaked already before age 2 years, and in the case of IA-2A between ages 2 and 5 years, whereas GADA-positive seroconversions demonstrated a steady rate up to between ages 10 and 15.5 years (20).

### Development of multipositivity

The proportion of multipositive children increased steadily over the first 15 years (20). However, only 4 children developed multipositivity for biochemical autoantibodies after age 10 years. Children with the high-risk HLA genotype demonstrated significantly more often multipositivity than those with moderate-risk genotypes (data not shown). The appearance of multiple biochemical autoantibodies conferred a 58% risk of developing clinical diabetes in the next 10 years (95% CI, 44%-71%). Intriguingly, the presence of ZnT8A in the first sample positive for multiple biochemical autoantibodies was associated with a higher 10-year risk of clinical diabetes (73.1%, n = 19; 95% CI, 54%-87%) than the absence of ZnT8A on this occasion (40.0%, n = 8; 95% CI, 22%-61%; *P* = .02). Season of birth contributed to age at multipositivity; participants born in the winter (December to February) tested more often multipositive for biochemical autoantibodies before age 2 years (67 vs 25%; *P* = .004) and tended to develop multipositivity for biochemical autoantibodies at a younger age than those born in other seasons (median, 1.5 vs 4.0 years; *P* = .06).

### Inverse seroconversions and fluctuating autoantibodies

Among the islet autoantibodies, IAA demonstrated most often inverse seroconversions and fluctuations (Table 2, [20]). No inverse seroconversions or fluctuations of ICA occurred in progressors. None of the children with multiple biochemical autoantibodies turned completely autoantibody negative by age 15.5 years, although one of them had only ICA in the last sample available. The rate of inverse IAA seroconversions was higher among children born in the fall (September to November) than among those born in other seasons (77 vs 43%; *P* = .03).

**Table 2.** Inverse seroconversions and fluctuating autoantibodies in study cohort

		Total	Progressors	Autoantibody-positive nonprogressors	*P* ^*g*^
		N (%)^*a*^	N (%)^*b*^	N (%)^*c*^	
Inverse seroconversions		124 (45.1)	13 (40.6)	111 (45.7)	.59
Fluctuating autoantibodies		110 (40.0)	12 (37.5)	98 (40.3)	.76
Multiple fluctuations of same reactivity		49 (17.8)	8 (25.0)	41 (16.9)	.26
Inverse seroconversions and/or fluctuations		180 (65.5)	21 (65.6)	159 (65.4)	.98
Inverse seroconversions and fluctuations		54 (19.6)	4 (12.5)	50 (20.6)	.28
		N (%)^*d*^	N (%)^*e*^	N (%)^*f*^	
Inverse seroconversion	ICA	77 (31.4)	0 (0)	77 (36.2)	< .001
	IAA	34 (49.3)	7 (25.9)	27 (64.3)	.002
	GADA	20 (29.0)	5 (20.0)	15 (34.1)	.22
	IA-2A	4 (9.3)	0 (0)	4 (22.2)	.01
	ZnT8A	9 (18.8)	3 (12.0)	6 (26.1)	.21
Fluctuating positivity	ICA	69 (28.2)	0 (0)	69 (32.4)	< .001
	IAA	25 (36.2)	7 (25.9)	18 (42.9)	.15
	GADA	14 (20.3)	1 (4.0)	13 (29.5)	.01
	IA-2A	6 (14.0)	2 (8.0)	4 (22.2)	.18
	ZnT8A	8 (16.7)	6 (24.0)	2 (8.7)	.16
		Median (range)			
Median age at inverse seroconversion, y	ICA	13.0 (1.0-15.4)	NA	NA	NA
	IAA	6.9 (1.3-13.7)	6.2 (1.8-10.6)	7.1 (1.3-13.7)	.48
	GADA	10.6 (1.5-14.3)	2.1 (1.5-9.7)	11.3 (5.7-14.3)	.006
	IA-2A	9.1 (2.8-10.0)	NA	NA	NA
	ZnT8A	4.0 (2.0-14.8)	4.0 (2.6–7.9)	3.9 (2.0-14.8)	.61
Median age at first AAB reappearance, y	ICA	12.6 (6.5-15.5)	NA	NA	NA
	IAA	8.1 (2.0-13.9)	6.3 (4.5-10.8)	8.1 (2.0-13.9)	.36
	GADA	8.4 (2.3-14.2)	14.2	8.2 (2.3-14.0)	.11
	IA-2A	8.1 (7.0-14.2)	10.8 (7.4-14.2)	8.1 (7.0-11.3)	.64
	ZnT8A	2.9 (1.8-9.0)	2.9 (1.8-4.3)	5.5 (2.0-9.0)	.51
Time from seroconversion to AAB disappearance, y	ICA	2.5 (0.5-9.1)	NA	NA	NA
	IAA	2.3 (0.5-13.4)	3.7 (1.3-8.0)	2.2 (0.5-13.4)	.69
	GADA	5.0 (0.5-9.8)	1.1 (0.9-5.7)	6.0 (0.5-9.8)	.06
	IA-2A	2.8 (1.0-5.5)	NA	NA	NA
	ZnT8A	2.2 (0.5-12.3)	2.2 (1.6-6.9)	3.1 (0.5-12.3)	.61
Time from seroconversion to first AAB fluctuation, y	ICA	4.0 (0.7-11.5)	NA	NA	NA
	IAA	3.8 (0.7-10.4)	3.5 (2.8-8.0)	4.1 (0.7-10.4)	.81
	GADA	3.5 (1.0-7.2)	7.2	3.5 (1.0-7.0)	.11
	IA-2A	5.2 (3.5-7.2)	6.8 (6.4-7.2)	4.1 (3.5-6.3)	.06
	ZnT8A	1.4 (1.0-10.2)	1.4 (1.0-3.8)	5.6 (1.0-10.2)	.74

Abbreviations: AAB, autoantibody; IAA, insulin autoantibodies; IA-2A islet antigen-2 autoantibodies; ICA, islet cell antibodies; GADA, glutamate decarboxylase autoantibodies; ZnT8A, zinc transporter 8 autoantibodies.

^*a*^Proportion of seroconverters (n = 275).

^*b*^Proportion of progressors with confirmed seroconversion (n = 32).

^*c*^Proportion of autoantibody-positive nonprogressors (n = 243).

^*d*^Proportion of study participants positive for the specific autoantibody reactivity (see Table 1).

^*e*^Proportion of progressors positive for the specific autoantibody reactivity (see Supplemental Table 2 [20]).

^*f*^Proportion of nonprogressors positive for the specific autoantibody reactivity (see Supplemental Table 2 [20]).

^*g*^ *P* = progressors vs autoantibody-positive nonprogressors.

### Effect of autoantibody titers on inverse seroconversions

Because IAA and ICA experienced inverse seroconversions and fluctuating patterns most frequently, we examined the association of their peak titers with the rate of inverse seroconversions. For every child who tested positive for ICA and/or IAA, the maximum autoantibody titer for each reactivity was used. The peak IAA titers were higher among those who remained IAA-positive (median 34.6 [n = 35] vs 11.0 [n = 34] relative units [RU]; *P *< .001). Similarly, the peak ICA titers were higher among those with persistent ICA (median 10.0 [n = 168] vs 5.0 [n = 77] JDFU; *P *< .001).

### Comparison of progressors to multipositive nonprogressors

We compared the progressors to nonprogressors who developed multipositivity. A higher proportion of progressors tested multipositive within a year from seroconversion, although this difference turned out to be statistically nonsignificant, when ICA was removed from the analysis (20). Without ICA in the analysis, the progressors tested more often positive for IAA at seroconversion especially before age 5 years, had more stable IAA positivity, and developed ZnT8A positivity at a younger age than the multipositive nonprogressors. The titers of ICA, IA-2A, and ZnT8A were significantly higher in progressors already several months before diagnosis when compared to matched multipositive controls (20).

### Primary autoantibodies at seroconversion

**Figure 3.**
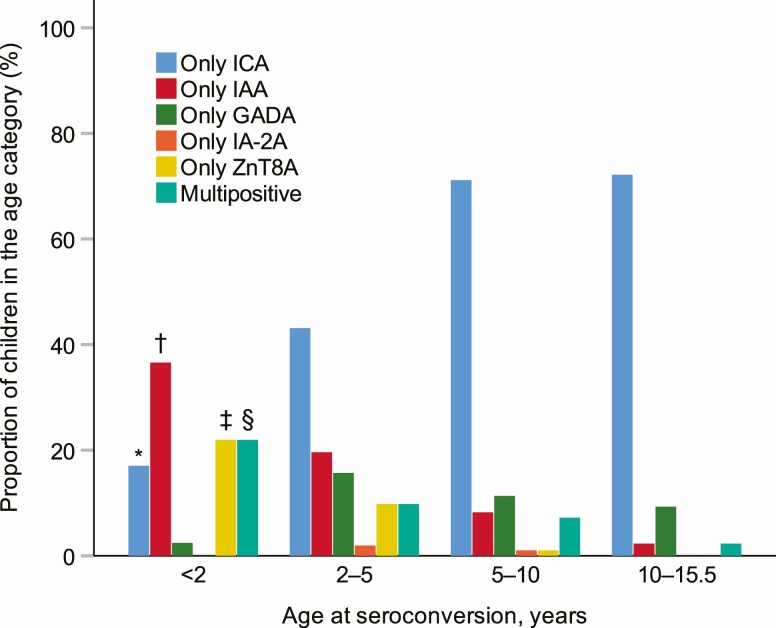
Autoantibody profiles at initial seroconversion in the age groups 0 to 2 (N = 41), 2 to 5 (N = 51), 5 to 10 (N = 97), and 10 to 15.5 (N = 86) years. *P less than .05 compared to all other groups. †*P* less than .05 0 to 2 vs 2 to 5, 0 to 2 vs 5 to 10, 0 to 2 vs 10 to 15.5, 2 to 5 vs 5 to 10, 2 to 5 vs 10 to 15.5 years. ‡*P* less than .05 0 to 2 vs 2 to 5, 0 to 2 vs 5 to 10, 0 to 2 vs 10 to 15.5, 2 to 5 vs 5 to 10 years. §*P* less than .05 0 to 2 vs 5 to 10, 0 to 2 vs 10 to 15.5 years.

Autoantibody profiles at initial seroconversion demonstrated distinct characteristics in different age groups (Fig. 3). Consistently with the age-related profiles, the primary autoantibodies present at seroconversion showed notable differences between the progressors and nonprogressors (Tables 3 and 4). The primary single autoantibody among progressors was predominantly IAA or ZnT8A, whereas in multipositive nonprogressors this was mainly GADA. ICA as a single autoantibody at seroconversion indicated nonprogressive autoimmunity regardless of the ICA titer.

**Table 3.** Primary autoantibody signatures in progressors, single autoantibody-positive nonprogressors, and nonprogressors who developed multipositivity, as well as among children with high- and moderate-risk human leukocyte antigen genotypes and in boys vs girls with islet cell antibodies included in the analysis

Primary autoantibodies	Total	Progressors	Multipositive nonprogressors	Single AAB-positive nonprogressors	*P* ^*c*^	*P* ^*d*^	*P* ^*e*^	*P* ^*f*^	High-risk HLA genotype	Moderate-risk HLA genotype	*P* ^*g*^	Boys	Girls	*P* ^*h*^
	n = 275	n = 32	n = 39	n = 204					n = 91	n = 184		n = 146	n = 129	
	N (%)													
Only ICA^*b*^	172 (62.5)	1 (3.1)	8 (20.5)	163 (79.9)	.19	<.001	< .001	< .001	50 (54.9)	122 (66.3)	.25	83 (56.8)	89 (69.0)	.06
Only IAA^*b*^	35 (12.7)	6 (18.8)	9 (23.1)	20 (9.8)	.27	.001	.006	.001	12 (13.2)	23 (12.5)	.69	21 (14.4)	14 (10.9)	.33
Only GADA^*b*^	28 (10.2)	0 (0)	13 (33.3)	15 (7.4)	.007	.29	< .001	.38	11 (12.1)	17 (9.2)	.34	20 (13.7)	8 (6.2)	.03
Only IA-2A^*b*^	2 (0.7)	0 (0)	0 (0)	2 (1.0)	NA	NA	NA	NA	0 (0)	2 (1.1)	.34	1 (0.7)	1 (0.8)	.95
Only ZnT8A^*a,b*^	15 (5.5)	7 (21.9)	4 (10.3)	4 (2.0)	0.003	< .001	.07	< .001	6 (6.6)	9 (4.9)	.55	7 (4.8)	8 (6.2)	.85
Multiple autoantibodies	23 (8.4)	18 (56.3)	5 (12.8)	0 (0)	< .001	NA	NA	< .001	12 (13.2)	11 (6.0)	.04	14 (9.6)	9 (7.0)	.44

Abbreviations: AAB, autoantibody; IAA, insulin autoantibodies; IA-2A islet antigen-2 autoantibodies; ICA, islet cell antibodies; GADA, glutamate decarboxylase autoantibodies; ZnT8A, zinc transporter 8 autoantibodies.

^*a*^ZnT8A data available at seroconversion for 12 progressors, 30 multipositive nonprogressors, 97 single autoantibody-positive nonprogressors, 46 children with high-risk HLA genotype, 93 children with moderate risk HLA genotype, 68 boys, and 71 girls.

^*b*^Multipositive individuals were excluded from comparisons of single autoantibody-positive signatures between groups.

^*c*^ *P* = progressors vs multipositive nonprogressors.

^*d*^ *P* = progressors vs single autoantibody-positive nonprogressors.

^*e*^ *P* = multipositive vs single autoantibody-positive nonprogressors.

^*f*^ *P* ^§^ = progressors vs all nonprogressors.

^*g*^ *P* = high vs moderate risk HLA genotype.

^*h*^ *P* = boys vs girls.

**Table 4.** Primary autoantibody signatures in progressors, single autoantibody-positive nonprogressors, and nonprogressors who developed multipositivity, as well as among children with high- and moderate-risk human leukocyte antigen genotypes and in boys vs girls without islet cell antibodies in the analysis

Primary autoantibodies	Total	Progressors	Multipositive nonprogressors	Single AAB-positive nonprogressors	*P* ^*c*^	*P* ^*d*^	*P* ^*e*^	*P* ^*f*^	High-risk HLA genotype	Moderate-risk HLA genotype	*P* ^*g*^	Boys	Girls	*P* ^*h*^
	n = 113	n = 32	n = 19	n = 62					n = 45	n = 68		n = 70	n = 43	
	N (%)													
Only IAA^*b*^	41 (36.3)	8 (25.0)	5 (26.3)	28 (45.2)	.58	.57	.24	.76	14 (31.1)	27 (39.7)	.51	26 (37.1)	15 (34.9)	.92
Only GADA^*b*^	37 (32.7)	3 (9.4)	9 (47.4)	25 (40.3)	.02	.03	.35	.02	16 (35.6)	21 (30.9)	.41	26 (37.1)	11 (25.6)	.32
Only IA-2A^*b*^	5 (4.4)	2 (6.3)	0 (0)	3 (4.8)	.49	.60	1.00	.28	1 (2.2)	4 (5.9)	.65	3 (4.3)	2 (4.7)	1.00
Only ZnT8A^*a,b*^	17 (15.0)	8 (25.0)	3 (15.8)	6 (9.7)	.15	.02	1.00	.02	7 (15.6)	10 (14.7)	.98	9 (12.9)	8 (18.6)	.55
Multiple autoantibodies	13 (11.5)	11 (34.4)	2 (10.5)	0 (0)	.10	NA	NA	< .001	7 (15.6)	6 (8.8)	.27	6 (8.6)	7 (16.3)	.21

^*a*^ZnT8A data available at biochemical autoantibody seroconversion for 18 progressors, 16 multipositive nonprogressors, 37 single autoantibody-positive nonprogressors, 29 children with high-risk HLA genotype, 42 children with moderate risk HLA genotype, 42 boys, and 29 girls.

^*b*^Multipositive individuals were excluded from comparisons of single autoantibody positive signatures between groups.

^*c*^ *P =* progressors vs multipositive nonprogressors.

^*d*^ *P* = progressors vs single autoantibody-positive nonprogressors.

^*e*^ *P* = multipositive vs single autoantibody-positive nonprogressors.

^*f*^ *P* = progressors vs all nonprogressors.

^*g*^ *P* = high vs moderate risk HLA genotype.

^*h*^ *P* = boys vs girls.

### Progression rate to overt type 1 diabetes

Although the number of progressors was relatively modest, we examined the associations of autoantibodies with the pace of disease progression. Among progressors, inverse seroconversions to IAA were associated with a prolonged delay from seroconversion to diagnosis when compared to persistent IAA positivity (8.2 [n = 7] vs 3.4 [n = 20] years; *P* = .01). At diagnosis, IAA positivity was more common among participants with the high-risk HLA genotype than among those with moderate-risk genotypes (75 [n = 15] vs 36% [n = 5]; *P* = .02). Those who tested IAA positive at diagnosis were younger at disease presentation than those who tested IAA negative on this occasion (median, 6.6 [n = 20] vs 11.4 [n = 11] years; *P* = .04), although there was no difference in age at seroconversion between these groups (1.6 [n = 20] vs 1.3 [n = 11] years; *P* = .52). Other autoantibodies at diagnosis were not associated with age at diagnosis (data not shown). Autoantibody data at the diagnosis of T1D are presented in Supplemental Table 6 (20).

### The role of islet cell autoantibodies in disease prediction

Sensitivities, specificities, and positive predictive values for predicting clinical T1D of individual autoantibodies and multipositivity with and without ICA are compiled in Supplemental Table 7 (20). We examined several thresholds for ICA positivity to assess whether the ICA titer affected the prediction of T1D. Among multipositive children, there were no ICA-negative children, whereas only 48 (68%) children had IAA, 52 (73%) GADA, 40 (56%) IA-2A, and 43 (61%) ZnT8A. Without ICA in the analysis, 21 (29.6%) multipositive children would have been missed. One of these children progressed to T1D and tested multipositive for biochemical autoantibodies at diagnosis. The initial ICA titer in this child was 10 JDFU. Most ICA-positive children remained negative for the biochemical autoantibodies, but high ICA titers increased the proportion of overlap (20). Most children with biochemical autoantibodies tested ICA positive in 15 years, and the titers of ICA were relatively high in these individuals.

## Discussion

Here we explored the natural dynamics of diabetes-associated autoantibodies by age 15 years in 1006 children with HLA-conferred susceptibility to T1D derived from the general population in Finland with the highest disease incidence in the world. We have previously reported 2- and 5-year follow-up data from the same cohort (13, 14). In the present study, we demonstrate that the primary autoantibody profiles are characteristic of age, and that ZnT8A may appear early in the disease process. In young children, β-cell autoimmunity often begins with IAA or ZnT8A positivity. GADA-initiated β-cell autoimmunity commonly appears later. ICA becomes the prevailing autoantibody toward puberty after the preschool years and if remaining as a single autoantibody does not have any value for predicting T1D. The biochemical autoantibodies show decreasing seroconversion rates with increasing age. There are no differences in seroconversion rates between boys and girls with aging. The proportion of multipositive children increases steadily over the first 15 years, although progression to biochemical autoantibody multipositivity rarely occurs after age 10 years. Among the islet autoantibodies, inverse seroconversions and fluctuations of IAA are most common. Accordingly, our findings are in line with previous characterizations of IAA appearing early but being rather unstable (9-14, 28, 29).

The strengths of this study are the general population-based study cohort and the regular autoantibody follow-up starting from birth, enabling close collaboration with the participating families. With respect to other extensive longitudinal follow-up studies, such as Type 1 Diabetes TrialNet or The Environmental Determinants of Diabetes in the Young (TEDDY), this is the first systematic prospective analysis of HLA-predisposed children that includes both ICA and ZnT8A in the screening repertoire in addition to the 3 other biochemical autoantibodies. Another advantage in the DIPP Study is the birth cohort–based ability to determine seroconversion age in all participants. The number of participants in the present study is relatively modest, but allows testing for statistical differences. Taking into account the limited size of the initial study population, the dropout rate was relatively high but tolerable considering the long 15-year follow-up. Many children were lost to follow-up during the last years of the study period, but at 10 years the dropout rate remained reasonable. Owing to the modest number of participants, the results should be interpreted cautiously. Corrections for multiple testing were not applied in the present data analysis, which might limit the interpretation of the results. The generalizability of the results is limited because all the study participants carry selected HLA-risk genotypes for T1D. However, these genotypes are found in the majority (62%) of children with T1D (18). Another limitation in this study is the lack of data on metabolic characteristics as an indicator for T1D risk.

As a novel finding, ZnT8A appeared commonly in very young children and in many cases as the first autoantibody even before IAA. Together with IA-2A, ZnT8A had the highest specificity for T1D. As the single first autoantibody at seroconversion, ZnT8A was strongly predictive for clinical disease. At diagnosis, ZnT8A were present in 66% of progressors, which is consistent with previous reports (25, 30). The titers of ZnT8A together with ICA and IA-2A started to rise in progressors already several months before diagnosis compared to matched multipositive controls. This is in line with earlier observations, stating that ZnT8A positivity at diagnosis occurs consistently with ICA and/or IA-2A, but less often with positivity for GADA or IAA (24, 30, 31). In general, ZnT8A might indicate an aggressive disease process. In β cells, ZnT8 is involved in insulin secretion, and is a disease-associated autoantigen of proinflammatory T cells (32). At diagnosis, ZnT8A positivity implies a higher risk of ketoacidosis and predicts an increased need for insulin after the diagnosis (30). Accordingly, our results suggest that ZnT8A appearing early in the disease process and as the primary autoantibody in young children might be a strong indicator of T1D. At diagnosis, ZnT8A positivity has been linked to the *HLA-DQ8* and *DQ6.4* haplotypes, but is inversely associated with the *DR3-DQ2/DR4-DQ8* genotype and the *DR3-DQ2* haplotype, which might be partly explained by differential binding of the DQ molecules to ZnT8 epitopes (25, 31, 33, 34). In the present study no association of ZnT8A with the HLA genotype was observed, probably because of the limited selection of HLA genotypes.

In young children, ZnT8A or IAA emerged commonly as the first autoantibody, whereas GADA-initiated islet autoimmunity increased in the preschool years and continued to appear steadily until the pubertal years. The different characteristics of β-cell autoimmunity initiated by either IAA or GADA have recently been studied (8-12). The 2 endotypes show different genetic associations (8, 9, 35), and the IAA-first endotype has been related to T1D-linked Coxsackie B1 infections, whereas primary GADA signature might arise from a different etiological background (36). In the present study, GADA as the first single autoantibody at seroconversion was rare among progressors, whereas it was characteristic of multipositive nonprogressors. In contrast, the primary autoantibody among progressors was mainly IAA or ZnT8A among those who were not multipositive at initial seroconversion. This highlights the existence of at least 2 paths of advanced islet autoimmunity and might reflect the fact that initial IAA reactivity peaks at a young age, whereas GADA appears more evenly over a series of years (10-12). In an earlier DIPP study, we compared the progression from seroconversion to clinical diabetes between children with either IAA or GADA as the first autoantibody but could not see any difference in the progression rate (9). The long follow-up of the whole cohort in the present study has especially increased the number of older children with GADA-initiated autoimmunity who have not yet progressed further. Moreover, the season of birth affects the pace of disease progression (15), and might contribute to age-related features of β-cell autoimmunity. In the present study, positivity for multiple biochemical autoantibodies appeared at a younger age among children born in the winter, whereas inverse seroconversions of IAA peaked among children born in the fall. The present observations together with previous reports support the idea of heterogeneity in the triggering events of β-cell autoimmunity that might be age-related, linked to the stage of immunological maturation, the strength of protection by maternal antibodies, and the exposure to seasonal environmental factors, including infections, which usually become more common in the preschool years as children start daycare. It is also intriguing to consider whether GADA might, in fact, reflect counteracting mechanisms against progressive autoimmunity. This idea is supported by the observations that the autoantigen for GADA, glutamic acid decarboxylase, is involved in the production of the anti-inflammatory neurotransmitter γ-aminobutyric acid (GABA), which has been shown to promote β-cell survival and replication, and to increase the human β-cell mass (37).

The loss of IAA reactivity has been associated with delayed disease progression in multipositive children (38). Although the number of progressors in the present study was modest, disease progression turned out to be slower when IAA disappeared before diagnosis. This finding raises questions as to why autoantibody production against insulin may be attenuated among individuals with delayed progression. First, when the loss of IAA positivity reflects events in humoral β-cell autoimmunity, cell-mediated autoimmunity against insulin may still continue. However, without the support of humoral immunity the disease process might slow down, which suggests that B cells are active players in the pathogenesis of T1D (39). Second, individuals with delayed progression may possess immunological mechanisms that are capable of hiding the β-cell autoantigens from the immune system. In fact, in several studies patients with T1D have been described in whom C-peptide production has been detectable after decades of clinical disease, suggesting that β cells were not completely destroyed (40, 41). In contrast to previous observations, reversion of GADA positivity did not affect the risk of clinical diabetes in the present study (42, 43). Reversions of multiple biochemical autoantibody positivity occurred rarely, which is in accordance with earlier reports and supports the idea that positivity for multiple biochemical autoantibodies might be seen as an early stage of T1D (44).

ICA is laborious to analyze and the assay is hard to standardize, thus replacing ICA with biochemical autoantibodies is enticing. Therefore we analyzed the dynamics of ICA in detail and evaluated the impact of excluding ICA from the autoantibody repertoire used for screening purposes. ICA had a high sensitivity to identify progressors to clinical disease, but in contrast to previously reported 5-year follow-up data, quite poor specificity compared to biochemical autoantibodies (14). This difference probably results from the steep increase in the ICA seroconversion rate after the preschool years. The significantly higher specificity and positive predictive value of positivity for multiple biochemical autoantibodies when compared to ICA positivity suggest that low-titer ICA can be replaced by positivity for multiple biochemical autoantibodies when screening for risk of T1D (20, 45, 46). However, if the threshold for ICA positivity is set at 10 JDFUs, ICA predict T1D and perform in the screening purposes similarly to the biochemical autoantibodies. Because the ICA method is biological, using pancreatic tissue, the method detects a wide variety of islet-specific antibodies and occasionally other cross-reactive antibodies in serum samples (47). Therefore, the ICA method can never be as specific as biochemical methods, and for the same reason it is more sensitive. In prediabetes screening, by determining the optimal JDF level of ICA that provides the best combination of sensitivity and specificity, the ICA method can provide something more than is available in the 4-biochemical autoantibody method set. The increasing ICA seroconversion rate toward adolescence is restricted to low-titer ICA. This type of ICA reactivity is not related to the risk of pediatric T1D, but may anticipate the fact that ICA positivity is commonly seen in adults (48). Removing low-titer ICA from the preclinical screening program in the general population would significantly affect the seroconversion age. This should be noted when explaining the risk of diabetes to physicians and the families of screened children.

Interestingly, none of the present progressors demonstrated transient or fluctuating ICA. This may reflect the fact that ICA reactivity is in part derived from reactions toward other islet autoantigens, and also yet undiscovered autoantigens may contribute to disease-associated ICA reactivity (47). In the present study, isolated low-titer ICA positivity occurred especially at initial seroconversion and the overlap of ICA with biochemical autoantibodies was restricted mostly to high ICA titers. Most children with biochemical autoantibodies tested positive for high-titer ICA with advancing age. The highest proportions of overlap with ICA were seen in children positive for IA-2A and ZnT8A.

Based on studies in the Finnish population, it was estimated at the beginning of the DIPP Study that approximately 7% of the high-risk and 2% to 3% of the moderate-risk children would develop T1D by age 15 years (18). The proportion of affected children in the high-risk (8.3%) and moderate-risk HLA groups (1.9%) match that prediction.

To conclude, the proportion of diabetes-associated autoantibodies in children HLA-predisposed to T1D increases steadily during the first 15 years of life, mostly because of low-titer ICA seroconversions. Biochemical autoantibodies and high-titer ICA show decreasing seroconversion rates with advancing age. The conspicuous differences in the age-related behavior of individual biochemical autoantibodies indicate that age and the stage of immunological maturation contribute substantially to the characteristics of islet autoimmunity. In young children, ZnT8A and IAA are the most common primary autoantibodies, but at preschool age GADA-driven islet autoimmunity increases and continues to appear steadily until the teenage years. The first-appearing autoantibody might reflect the events driving the disease process toward overt disease. The fluid state of autoantibodies, especially IAA, might impart the risk for T1D. These findings provide a valuable framework for understanding the disease heterogeneity and for personalizing the efforts to predict T1D. Further investigation into the age-dependent paths of humoral islet autoimmunity might provide mechanistic insights into the disease process.

## Data Availability

The data sets generated during and/or analyzed during the present study are not publicly available but are available from the corresponding author on reasonable request.

## Abbreviations

AAB: autoantibody
DIPP: Type 1 Diabetes Prediction and Prevention Study
GADA: glutamic acid decarboxylase autoantibodies
HLA: human leukocyte antigen
IAA: insulin autoantibodies
IA-2A: islet antigen-2 autoantibodies
ICA: islet cell antibodies
JDFU: Juvenile Diabetes Foundation units
T1D: type 1 diabetes
ZnT8A: zinc transporter 8 autoantibodies

## Additional Information

***Disclosure Summary:*** The authors have nothing to disclose.
